# Fifteen microsatellite markers for the Appalachian rockcap fern, *Polypodium appalachianum* (Polypodiaceae), and its relatives

**DOI:** 10.1002/aps3.1195

**Published:** 2018-11-08

**Authors:** Vishnupriya Kasireddy, Erika Mitchell, Zack E. Murrell, Emily L. Gillespie

**Affiliations:** ^1^ Department of Biological Sciences Marshall University One John Marshall Drive Huntington West Virginia 25755 USA; ^2^ Better Life Laboratories 293 George Road East Calais Vermont 05650 USA; ^3^ Department of Biology Appalachian State University 572 Rivers Street Boone North Carolina 28608 USA; ^4^ Department of Biological Sciences Butler University 4600 Sunset Avenue Indianapolis Indiana 46208 USA

**Keywords:** Polypodiaceae, *Polypodium appalachianum*, *Polypodium virginianum*, *Polypodium vulgare* complex, species boundaries

## Abstract

**Premise of the Study:**

Microsatellite markers were developed for *Polypodium appalachianum* (Polypodiaceae) to facilitate investigation of species boundaries between *P. appalachianum* and its putative hybrid, *P. virginianum*, and potentially among other members of the Miocene‐age *P. vulgare* species complex.

**Methods and Results:**

Forty‐eight primer pairs were designed from Illumina data and screened for successful amplification. Sixteen pairs were genotyped and evaluated for variability within and among three populations in North Carolina, Vermont, and New Hampshire. Twelve of these primer pairs were reliable and polymorphic, exhibiting one to 10 alleles per locus. Cross‐species amplification experiments were conducted for *P. virginianum* and four additional close relatives from the *P. vulgare* complex in order to maximize information about likely utility within a phylogenetic context.

**Conclusions:**

These microsatellite markers will be useful in population genetics and species boundaries studies of *P. appalachianum* and *P. virginianum*, and likely in other species within the *P. vulgare* complex.

The *Polypodium vulgare* complex (Polypodiaceae) is a group of Northern Hemisphere fern species once recognized as a single species, *P. vulgare* L. During the 20th century, this single species was eventually split into 16 species and numerous sterile hybrids based on evidence from cytological, hybridization, geographical, and morphological studies (e.g., Haufler et al., 1995). Ten of these species have been described as diploids, and they form four lineages that are supported by multiple molecular markers and morphological evidence. Six more species are considered allopolyploids, derived from hybridization between two or more diploid progenitor species, likely within the last 15 million years (Bryan and Soltis, 1987; Haufler et al., 1995; Sigel et al., 2014). The patterns and mechanisms of evolution at the population level are not well understood, and no microsatellite markers have been previously developed for the *P. vulgare* complex, impeding progress toward understanding these recently derived species.

One primarily North American lineage (‘Clade A’ in Sigel et al., 2014) is composed of three diploid species (*P. appalachianum* Haufler & Windham, *P. amorphum* Suksd., and *P. sibiricum* Sipliv.) and two allopolyploid species (*P. saximontanum* Windham and *P. virginianum* L.). The Appalachian rockcap fern, *P. appalachianum*, is distributed across the Appalachian Mountains, from Ontario to northern Georgia, and is a diploid (2*n* = 74; Haufler and Wang, 1991). The common rockcap fern, *P. virginianum*, is a tetraploid (2*n* = 148; Haufler et al., 1993) derived from hybridization between *P. appalachianum* and *P. sibiricum. Polypodium virginianum* overlaps the distribution of *P. appalachianum* in the Appalachian Mountains and is also found in the Ozark Mountains and the Great Lakes region (Haufler and Wang, 1991). *Polypodium amorphum* and *P. saximontanum* occur in the western United States, whereas *P. sibiricum* is distributed across northern Canada into Alaska and northern Asia. All species in this lineage tend to be found on rocky outcrops or cliffs, and *P. appalachianum* and *P. virginianum* are nearly morphologically indistinguishable where they co‐occur (Haufler et al., 1993). Development of microsatellite markers focused on the diploid *P. appalachianum* will yield a novel genetic tool for use in evolutionary studies within the *P. appalachianum–P. virginianum* complex, within the broader Clade A complex, and possibly across the *P. vulgare* complex.

## METHODS AND RESULTS

DNA was extracted from one individual of *P. appalachianum* using a modified cetyltrimethylammonium bromide (CTAB) approach (Doyle and Doyle, 1987) (Appendix 1). The West Virginia University Genomics Core Facility (Morgantown, West Virginia, USA) conducted 2 × 250 paired‐end Illumina sequencing on a MiSeq 600 cycle v3 microsatellite sequencing library (Illumina, San Diego, California, USA). A total of 1,985,528 raw sequence reads (GenBank Sequence Read Archive accession no. SRP148824) were trimmed of vectors and low‐quality sequence using the BBDuk 1.0 Geneious 9.1.7 plugin (Kearse et al., 2012; Biomatters Ltd., Auckland, New Zealand). MSATCOMMANDER 1.0.8 (Faircloth, 2008) was used to query the Illumina reads with default settings, except that mononucleotide repeats were omitted, minimum primer size was 20 bp, maximum primer GC content was 50%, and a PIG‐tail sequence (GTTT) (Brownstein et al., 1996) was appended to each forward primer. Unique DNA suitable for primer design flanked 2000 microsatellites (out of 11,528 discovered by MSATCOMMANDER).

Forty‐eight primer pairs, prioritizing motif diversity and annealing temperature (*T*_a_) difference ≤1°C, were screened in seven *P. appalachianum* individuals (Appendix 1) extracted using a QIAGEN Plant Mini Kit (QIAGEN, Hilden, Germany). PCRs were prepared in a 10‐μL reaction consisting of 1× GoTaq Flexi Buffer, 2.5 mM of MgCl_2_, 800 μM of dNTPs, 0.5 μM of each primer, 0.5 units of GoTaq Flexi DNA Polymerase (Promega Corporation, Madison, Wisconsin, USA), and ~20 ng of DNA. A touchdown program (*T*_a_ = 68–55°C) on a T100 thermal cycler (Bio‐Rad, Hercules, California, USA) was employed. Initial denaturation was 94°C for 5 min; followed by 13 cycles of 45 s at 94°C, 2 min at touchdown temperature, and 1 min at 72°C; followed by 24 cycles of 45 s at 94°C, 1 min at 55°C, and 1 min at 72°C; and followed by 5 min at 72°C. Amplicons were visualized on a 1% agarose gel in 1× TBE and scored for the presence or absence (and approximate size) of strong, single amplicons in the anticipated size range. Sixteen primer pairs (Table 1) produced amplicons worthy of development, and these were screened for polymorphisms in 68 individuals from three populations from North Carolina, New Hampshire, and Vermont (Appendix 1).

**Table 1:** Characteristics of 16 microsatellite primer pairs screened for *Polypodium appalachianum*

Locus	Primer sequences (5′–3′)^a^	Fluorescent dye	Repeat motif	*T*_a_ (°C)	Allele size range (bp)	GenBank accession no.
PAPP001	F: TTACATGCCGCAACTTGACC	PET	(AAAG)_6_	59.01	286–298	MH397691
	R: GTTTACTAGCAGATTGTCTCATAGGC					
PAPP004	F: AGGTACTTGGAAGAGGATGGTC	6‐FAM	(AAG)_8_	59.6	97	MH397692
	R: GTTTAGCCATGCCATTCAAATCCC					
PAPP013	F: ATATCAACTGGCCGCAACTC	VIC	(AC)_8_	59.2	418–420	MH397693
	R: GTTTGATCCCTTTGTGCTAGGC					
PAPP026	F: ATAAGCATCAACCGAGCGAC	NED	(ACCCGC)_6_	59.0	162–198	MH397694
	R: GTTTGCACACTCCAGATTCCC					
PAPP027	F: AGGTAAATGCTTCGGATGCG	NED	(ACCTCC)_7_	58.8	162–186	MH397695
	R: GTTTCCAAGACTTGAAGCGTTGTC					
PAPP028	F: CAATTTGCAGCAAGAAGGGC	6‐FAM	(AG)_8_	59.1	197–203	MH397696
	R: GTTTAACATGGTTTGCGTCCTCC					
PAPP029	F: TCTACTTAGCACCTCCCATACC	NED	(AG)_12_	59.1	245–293	MH397697
	R: GTTTCTCCGTTTCGAGCAGAC					
PAPP030	F: TGAAAGTGAAATTGGGACGCC	PET	(AG)_15_	59.8	289–315	MH397698
	R: GTTTGACGTGTTGACTTCCGTG					
PAPP032	F: AAGTCCCTCCATATCCATCTCC	PET	(AG)_20_	59.6	264–266	MH397699
	R: GTTTGCTCCCATTCGACGTTG					
PAPP038	F: TCATTGCTCAACCCTACACC	6‐FAM	(AGAT)_6_	58.2	187	MH397700
	R: GTTTGACTCTCATCTAGGTACGC					
PAPP039	F: GCCTTGACAATTGCAGCTTG	NED	(AGC)_8_	58.9	258–276	MH397701
	R: GTTTACTTGGGATGGATGAGCATG					
PAPP040	F: ACGCACAAGAAATTTCCTTGC	VIC	(AT)_8_	58.9	82	MH397702
	R: GTTTCAAGGCTACGAAGATGGC					
PAPP042	F: TGTAATGGCCAAGGTATGCC	6‐FAM	(AT)_12_	58.8	NA^b^	MH397703
	R: GTTTCAGCCCTTTGTGGCATAG					
PAPP046	F: AACTAAGGGCCGGAACTTTG	VIC	(ATC)_8_	58.5	293–299	MH397704
	R: GTTTACTCATATCAACACCAGGCC					
PAPP047	F: ACCTCCAATGTCAACCAAAGAG	VIC	(ATC)_11_	58.9	381–408	MH397705
	R: GTTTCATGGTGGTTCTCGTATGG					
PAPP048	F: AGGGAAAGTATGTCCGCTTG	PET	(ATC)_13_	59.1	283–292	MH397706
	R: GTTTCAGCCACTTCATCATGCC					

Note

*T*_a_ = annealing temperature.

a PIG‐tail sequence is underlined on reverse primers.

b Marker PAPP042 failed genotyping and therefore no size range information is available.

PCR reaction protocols from the amplification screen were maintained for the polymorphism screen, except that the forward primer was reduced to 0.25 μM and replaced with 0.25 μM of M13 primer (5′‐CACGACGTTGTAAAACGAC‐3′) labeled with 6‐FAM, VIC, NED, or PET (Life Technologies, Grand Island, New York, USA). Differently labeled, pool‐plexed PCR products (2 μL total) along with a GeneScan 500 LIZ Size Standard (Life Technologies) were genotyped on an ABI 3730xl DNA Analyzer at the Georgia Genomics Facility (Athens, Georgia, USA). Resulting chromatograms were manually scored using Geneious 11.0.3. We employed explicit criteria for determining genotypes: (1) peaks were well‐defined and at least 3000 relative fluorescence units (RFUs) and (2) heterozygotes were called only if the second peak's RFU was ≥90% of the first peak. Genotypes were analyzed using GenAlEx 6.503 (Peakall and Smouse, 2006, 2012) to obtain standard descriptive statistics, to test for Hardy–Weinberg equilibrium (HWE) deviations, to examine the ability of the markers to distinguish among populations, and to determine the likelihood of clonality within each population.

Fifteen loci revealed chromatograms with no more than two peaks, indicating diploidy. One marker (PAPP042) genotyped poorly and was not developed further. Twelve polymorphic loci exhibited one to 10 alleles across three populations (mean 2.65) (Table 2). Observed heterozygosity ranged from 0.000–0.524 (mean 0.109). Eight loci (67%) failed to meet the expectations of HWE in at least one population. Of these, two loci failed to meet HWE assumptions in all sampled populations. Genetic distance followed by principal coordinates analysis (Orloci, 1978) demonstrated that the 12 polymorphic loci distinguish among populations, with the first three axes explaining 32.97% of the variation. A multilocus matches analysis (Peakall and Smouse, 2006, 2012) of 12 polymorphic loci discovered no identical individuals within or among populations, suggesting little or no clonality.

**Table 2:** Descriptive statistics for 15 microsatellite loci in *Polypodium appalachianum*.^a^^,^ b

Locus	Alleghany Co., NC (*N* = 24)				Washington Co., VT (*N* = 22)				Merrimack Co., NH (*N* = 22)			
	*A*	*H*_o_	*H*_e_	HWE^c^	*A*	*H*_o_	*H*_e_	HWE^c^	*A*	*H*_o_	*H*_e_	HWE^c^
PAPP001	3	0.238	0.461	**	3	0.050	0.566	***	3	0.227	0.530	NS
PAPP004	1	0.000	0.000	M	1	0.000	0.000	M	1	0.000	0.000	M
PAPP013	2	0.217	0.194	NS	1	0.000	0.000	M	1	0.000	0.000	M
PAPP026	3	0.208	0.258	***	4	0.524	0.618	NS	1	0.000	0.000	M
PAPP027	2	0.000	0.087	***	1	0.000	0.000	M	1	0.000	0.000	M
PAPP028	1	0.000	0.000	M	1	0.000	0.000	M	2	0.091	0.087	NS
PAPP029	10	0.217	0.811	***	4	0.053	0.729	***	6	0.227	0.750	***
PAPP030	5	0.176	0.465	**	5	0.111	0.710	***	7	0.000	0.705	***
PAPP032	2	0.286	0.308	NS	2	0.143	0.210	NS	2	0.182	0.434	**
PAPP038	1	0.000	0.000	M	1	0.000	0.000	M	1	0.000	0.000	M
PAPP039	4	0.250	0.648	***	3	0.136	0.129	NS	6	0.048	0.407	***
PAPP040	1	0.000	0.000	M	1	0.000	0.000	M	1	0.000	0.000	M
PAPP046	2	0.043	0.122	**	2	0.048	0.046	NS	1	0.000	0.000	M
PAPP047	3	0.067	0.238	***	3	0.071	0.482	***	2	0.182	0.165	NS
PAPP048	2	0.524	0.387	NS	2	0.143	0.133	NS	2	0.455	0.351	NS
Mean	2.80	0.148	0.265		2.47	0.085	0.236		2.53	0.094	0.228	

Note

*A* = number of alleles detected across all individuals; *H*_e_ = expected heterozygosity; *H*_o_ = observed heterozygosity; HWE = Hardy–Weinberg equilibrium; *N* = number of individuals sampled; NC = North Carolina; NH = New Hampshire; VT = Vermont.

Location and voucher information are given in Appendix 1.

A sixteenth marker, PAPP042, did not genotype well enough to analyze.

Asterisks (*) indicate statistically significant deviation from HWE (*P < 0.05, **P < 0.01, ***P < 0.001). M = monomorphic marker; NS = not statistically significant. PAPP004, PAPP038, and PAPP040 are monomorphic in all three populations.

Cross‐amplification of 16 primer pairs was conducted within a phylogenetic context, following Sigel et al. (2014). Five individuals of *P. virginianum*, two of *P. amorphum*, and one of *P. sibiricum* (i.e., all members of ‘Clade A’), as well as three representatives each of *P. glycyrrhiza* D. C. Eaton and *P. scouleri* Hook. & Grev. (members of more distant *P. vulgare* complex clades) were included. Multiple individuals of the more distant relatives were included when possible because we were limited to fairly old herbarium material. Ten primer pairs amplified well in at least four related species. Fourteen markers amplified well in *P. virginianum*, and all markers that were variable in *P. appalachianum* were also variable in *P. virginianum* (Table 3).

**Table 3:** Cross‐amplification of 16 primer pairs in additional representatives of the *Polypodium vulgare* complex.^a^^,^ b

Species	*P. virginianum* (*N* = 5)	*P. amorphum* (*N* = 2)	*P. sibiricum* (*N* = 1)	*P. glycyrrhiza* (*N* = 3)	*P. scouleri* (*N* = 3)
PAPP001	286–298	294	—	290	286–290
PAPP004	97	97	—	97	97
PAPP013	418–420	420	—	418–420	418–420
PAPP026	186–192	168	168	162–168	168
PAPP027	162–174	162	168	156–168	162–168
PAPP028	197–203	201	—	201	203
PAPP029	251–287	264	—	—	—
PAPP030	—	—	—	—	—
PAPP032	264–266	264–266	—	262–266	262–266
PAPP038	187	—	187	—	—
PAPP039	258–279	261	—	258–261	264
PAPP040	82	82	—	82	82
PAPP042	—	—	—	—	—
PAPP046	293–299	293	—	293	—
PAPP047	387–411	381	—	384	384
PAPP048	286–295	283–286	—	283–289	—

Note

— = no observable amplification; *N* = number of individuals sampled.

a Location and voucher information for outgroup representatives are given in Appendix 1.

b Allele size range is given if the marker was variable and multiple individuals were sampled.

## CONCLUSIONS

The markers reported here will be useful in population studies within *P. appalachianum*, successfully differentiating among three populations spanning the species’ distribution, and for distinguishing between *P. appalachianum* and *P. virginianum*. Importantly, we demonstrate the value in testing markers in more phylogenetically distant species in order to maximize the utility of markers to the plant evolution and systematics community.

## DATA ACCESSIBILITY

The raw sequence reads are deposited in the National Center for Biotechnology Information (NCBI; GenBank Sequence Read Archive accession no. SRP148824). Sequence information for the developed primers has been deposited to NCBI; accession numbers are provided in Table 1.

## Appendix 1 Voucher information for *Polypodium* individuals included in this study.

**Table:**

Species	Voucher (Herbarium)	Geographic coordinates		Elevation (m)	State/Province	County/unit	*N*
		Latitude	Longitude				
*P. appalachianum* Haufler & Windham	Gillespie 16‐001 (MUHW)^a^	36.30	−81.72	1565	North Carolina	Watauga	1
*P. appalachianum*	Gillespie 17‐004 (MUHW)^b^	36.55	−81.32	792	North Carolina	Alleghany	24
*P. appalachianum*	Mitchell 471 (MUHW)^b^	44.46	−72.40	434	Vermont	Washington	22
*P. appalachianum*	Mitchell 511 (MUHW)^b^	43.08	−71.57	159	New Hampshire	Merrimack	22
*P. virginianum* L.	Coovert 14 (MUHW)^c^	38.30	−82.35	187	West Virginia	Wayne	1
*P. virginianum*	Kilpatrick 9c (MUHW)^c^	39.02	−79.68	1052	West Virginia	Tucker	1
*P. virginianum*	Strickland 208 (MUHW)^c^	38.50	−81.42	293	West Virginia	Kanawha	1
*P. virginianum*	Wyckle 3 (MUHW)^c^	37.92	−80.29	604	West Virginia	Greenbrier	1
*P. virginianum*	Dickson 5 (MUHW)^c^	38.61	−82.60	239	Ohio	Lawrence	1
*P. amorphum* Suksd.	Legler 14297 (WTU)^c^	48.99	−121.86	1569	Washington	Whatcom	1
*P. amorphum*	Legler 12062 (WTU)^c^	47.39	−123.40	568	Washington	Mason	1
*P. sibiricum* Sipliv.	Legler 1292 (WTU)^c^	48.00	142.53	39	Russia	Sakhalin	1
*P. glycyrrhiza* D. C. Eaton	Legler 14242 (WTU)^c^	48.02	−121.64	997	Washington	Snohomish	1
*P. glycyrrhiza*	Johnson NWCA16 (WTU)^c^	47.50	−121.81	133	Washington	King	1
*P. glycyrrhiza*	Montgomery 01‐04 (KHD)^c^	55.20	−131.38	5	Alaska	Ketchikan Gtwy	1
*P. scouleri* Hook. & Grev.	Walker 741 (WTU)^c^	47.31	−124.27	4	Washington	Grays Harbor	1
*P. scouleri*	Camp 1763 (WTU)^c^	46.29	−124.06	27	Washington	Pacific	1
*P. scouleri*	Yeatts 3593 (KHD)^c^	48.67	−124.86	1771	British Columbia	Vancouver Island	1

Notes

KHD = Kathryn Kalmbach Herbarium (Denver Botanic Garden); MUHW = Marshall University Herbarium; *N* = number of individuals; WTU = Burke Museum (University of Washington).

a Voucher for Illumina sequencing.

b Voucher for marker development (separate collection effort).

c Voucher for cross‐amplification.
